# Supplementary material for: Integrated Bioinformatics Analysis Reveals Marker Genes and Potential Therapeutic Targets for Pulmonary Arterial Hypertension
Source: Genes (Basel). 2021 Aug 28;12(9):1339. doi: 10.3390/genes12091339 (PMC8467453; doi:10.3390/genes12091339)
Supplement: Supplementary file 1 [file genes-12-01339-s001.zip › genes-1315395-supplementary.pdf]

Table S1 GSE113439 sample information

Group	GEO Accession	Organism	Platform ID	Disease state	Tissue
Pulmonary arterial hypertension patient 1	GSM3106326	Homo sapiens	GPL6244	idiopathic PAH patient	Lung
Pulmonary arterial hypertension patient 2	GSM3106327	Homo sapiens	GPL6244	patient with PAH and CHD	Lung
Pulmonary arterial hypertension patient 3	GSM3106328	Homo sapiens	GPL6244	patient with PAH and CTD	Lung
Pulmonary arterial hypertension patient 4	GSM3106329	Homo sapiens	GPL6244	patient with PAH and CHD	Lung
Pulmonary arterial hypertension patient 5	GSM3106330	Homo sapiens	GPL6244	idiopathic PAH patient	Lung
Pulmonary arterial hypertension patient 6	GSM3106331	Homo sapiens	GPL6244	idiopathic PAH patient	Lung
Pulmonary arterial hypertension patient 7	GSM3106332	Homo sapiens	GPL6244	patient with PAH and CTD	Lung
Pulmonary arterial hypertension patient 8	GSM3106333	Homo sapiens	GPL6244	patient with PAH and CHD	Lung
Pulmonary arterial hypertension patient 9	GSM3106334	Homo sapiens	GPL6244	patient with PAH and CTD	Lung
Pulmonary arterial hypertension patient 10	GSM3106335	Homo sapiens	GPL6244	patient with PAH and CTD	Lung
Pulmonary arterial hypertension patient 11	GSM3106336	Homo sapiens	GPL6244	idiopathic PAH patient	Lung

Pulmonary arterial hypertension patient 12	GSM3106337	Homo sapiens	GPL6244	idiopathic PAH patient	Lung
Pulmonary arterial hypertension patient 13	GSM3106338	Homo sapiens	GPL6244	CTEPH patient	Lung
Pulmonary arterial hypertension patient 14	GSM3106339	Homo sapiens	GPL6244	idiopathic PAH patient	Lung
Pulmonary arterial hypertension patient 15	GSM3106340	Homo sapiens	GPL6244	patient with PAH and CHD	Lung
Normal control 1	GSM3106341	Homo sapiens	GPL6244	normal control	Lung
Normal control 2	GSM3106342	Homo sapiens	GPL6244	normal control	Lung
Normal control 3	GSM3106343	Homo sapiens	GPL6244	normal control	Lung
Normal control 4	GSM3106344	Homo sapiens	GPL6244	normal control	Lung
Normal control 5	GSM3106345	Homo sapiens	GPL6244	normal control	Lung
Normal control 6	GSM3106346	Homo sapiens	GPL6244	normal control	Lung
Normal control 7	GSM3106347	Homo sapiens	GPL6244	normal control	Lung
Normal control 8	GSM3106348	Homo sapiens	GPL6244	normal control	Lung
Normal control 9	GSM3106349	Homo sapiens	GPL6244	normal control	Lung

Normal control 10	GSM3106350	Homo sapiens	GPL6244	normal control	Lung
Normal control 11	GSM3106351	Homo sapiens	GPL6244	normal control	Lung

Table S2 GSE53408 sample information

Group	GEO Accession	Organism	Platform ID	Disease state	Tissue
PAH1	GSM1290988	Homo sapiens	GPL6244	pulmonary arterial hypertension (PAH) patient	lung
PAH2	GSM1290989	Homo sapiens	GPL6244	pulmonary arterial hypertension (PAH) patient	lung
PAH3	GSM1290990	Homo sapiens	GPL6244	pulmonary arterial hypertension (PAH) patient	lung
PAH4	GSM1290991	Homo sapiens	GPL6244	pulmonary arterial hypertension (PAH) patient	lung
PAH5	GSM1290992	Homo sapiens	GPL6244	pulmonary arterial hypertension (PAH) patient	lung
PAH6	GSM1290993	Homo sapiens	GPL6244	pulmonary arterial hypertension (PAH) patient	lung
PAH7	GSM1290994	Homo sapiens	GPL6244	pulmonary arterial hypertension (PAH) patient	lung
PAH8	GSM1290995	Homo sapiens	GPL6244	pulmonary arterial hypertension (PAH) patient	lung
PAH9	GSM1290996	Homo sapiens	GPL6244	pulmonary arterial hypertension (PAH) patient	lung
PAH10	GSM1290997	Homo sapiens	GPL6244	pulmonary arterial hypertension (PAH) patient	lung
PAH11	GSM1290998	Homo sapiens	GPL6244	pulmonary arterial hypertension (PAH) patient	lung

PAH12	GSM1290999	Homo sapiens	GPL6244	pulmonary arterial hypertension (PAH) patient	lung
Control 1	GSM1291000	Homo sapiens	GPL6244	normal control	lung
Control 2	GSM1291001	Homo sapiens	GPL6244	normal control	lung
Control 3	GSM1291002	Homo sapiens	GPL6244	normal control	lung
Control 4	GSM1291003	Homo sapiens	GPL6244	normal control	lung
Control 5	GSM1291004	Homo sapiens	GPL6244	normal control	lung
Control 6	GSM1291005	Homo sapiens	GPL6244	normal control	lung
Control 7	GSM1291006	Homo sapiens	GPL6244	normal control	lung
Control 8	GSM1291007	Homo sapiens	GPL6244	normal control	lung
Control 9	GSM1291008	Homo sapiens	GPL6244	normal control	lung
Control 10	GSM1291009	Homo sapiens	GPL6244	normal control	lung
Control 11	GSM1291010	Homo sapiens	GPL6244	normal control	lung

Table S3 GSE15197 sample information

Group	GEO Accession	Organism	Platform ID	Disease state	Tissue
control, biological rep1	GSM379316	Homo sapiens	GPL6480	normal control	lung
control, biological rep2	GSM379317	Homo sapiens	GPL6480	normal control	lung
control, biological rep3	GSM379318	Homo sapiens	GPL6480	normal control	lung
control, biological rep4	GSM379319	Homo sapiens	GPL6480	normal control	lung
control, biological rep5	GSM379320	Homo sapiens	GPL6480	normal control	lung
control, biological rep6	GSM379321	Homo sapiens	GPL6480	normal control	lung
control, biological rep7	GSM379322	Homo sapiens	GPL6480	normal control	lung
control, biological rep8	GSM379323	Homo sapiens	GPL6480	normal control	lung
control, biological rep9	GSM379324	Homo sapiens	GPL6480	normal control	lung
control, biological rep10	GSM379325	Homo sapiens	GPL6480	normal control	lung
control, biological rep11	GSM379326	Homo sapiens	GPL6480	normal control	lung

control, biological rep12	GSM379327	Homo sapiens	GPL6480	normal control	lung
control, biological rep13	GSM379328	Homo sapiens	GPL6480	normal control	lung
IPF with PH, biological rep1	GSM379329	Homo sapiens	GPL6480	pulmonary hypertension secondary to idiopathic pulmonary fibrosis.	lung
IPF with PH, biological rep2	GSM379330	Homo sapiens	GPL6480	pulmonary hypertension secondary to idiopathic pulmonary fibrosis.	lung
IPF with PH, biological rep3	GSM379331	Homo sapiens	GPL6480	pulmonary hypertension secondary to idiopathic pulmonary fibrosis.	lung
IPF with PH, biological rep4	GSM379332	Homo sapiens	GPL6480	pulmonary hypertension secondary to idiopathic pulmonary fibrosis.	lung
IPF with PH, biological rep5	GSM379333	Homo sapiens	GPL6480	pulmonary hypertension secondary to idiopathic pulmonary fibrosis.	lung
IPF with PH, biological rep6	GSM379334	Homo sapiens	GPL6480	pulmonary hypertension secondary to	lung

				idiopathic pulmonary fibrosis.	
IPF with PH, biological rep7	GSM379335	Homo sapiens	GPL6480	pulmonary hypertension secondary to idiopathic pulmonary fibrosis.	lung
IPF with PH, biological rep8	GSM379336	Homo sapiens	GPL6480	pulmonary hypertension secondary to idiopathic pulmonary fibrosis.	lung
PAH, biological rep1	GSM379337	Homo sapiens	GPL6480	pulmonary arterial hypertension.	lung
PAH, biological rep2	GSM379338	Homo sapiens	GPL6480	pulmonary arterial hypertension.	lung
PAH, biological rep3	GSM379339	Homo sapiens	GPL6480	pulmonary arterial hypertension.	lung
PAH, biological rep4	GSM379340	Homo sapiens	GPL6480	pulmonary arterial hypertension.	lung
PAH, biological rep5	GSM379341	Homo sapiens	GPL6480	pulmonary arterial hypertension.	lung
PAH, biological rep6	GSM379342	Homo sapiens	GPL6480	pulmonary arterial hypertension.	lung
PAH, biological rep7	GSM379343	Homo sapiens	GPL6480	pulmonary arterial hypertension.	lung
PAH, biological rep8	GSM379344	Homo sapiens	GPL6480	pulmonary arterial hypertension.	lung

PAH, biological rep9	GSM379345	Homo sapiens	GPL6480	pulmonary arterial hypertension.	lung
PAH, biological rep10	GSM379346	Homo sapiens	GPL6480	pulmonary arterial hypertension.	lung
PAH, biological rep11	GSM379347	Homo sapiens	GPL6480	pulmonary arterial hypertension.	lung
PAH, biological rep12	GSM379348	Homo sapiens	GPL6480	pulmonary arterial hypertension.	lung
PAH, biological rep13	GSM379349	Homo sapiens	GPL6480	pulmonary arterial hypertension.	lung
PAH, biological rep14	GSM379350	Homo sapiens	GPL6480	pulmonary arterial hypertension.	lung
PAH, biological rep15	GSM379351	Homo sapiens	GPL6480	pulmonary arterial hypertension.	lung
PAH, biological rep16	GSM379352	Homo sapiens	GPL6480	pulmonary arterial hypertension.	lung
PAH, biological rep17	GSM379353	Homo sapiens	GPL6480	pulmonary arterial hypertension.	lung
PAH, biological rep18	GSM379354	Homo sapiens	GPL6480	pulmonary arterial hypertension.	lung

Table S4 Primer information

Gene	Forward	Reverse
Cdc5l	5′-CTTTCCCGTCGCCGCTTT-3′	5′-CTTTCAGAATTTCATCCTCGGTGT-3′
Ddx46	5′-ACAACAGAAGGCTGAGGGAAA-3′	5′ -TCGTTAGCCAAGGCCTGTTC-3′
18s	5′-ACCGCAGCTAGGAATAATGGA-3′	5′-GCCTCAGTTCCGAAAACCA-3′
