# Supplementary figures and images for: Integrated Bioinformatics Analysis Reveals Marker Genes and Potential Therapeutic Targets for Pulmonary Arterial Hypertension
Source: Genes (Basel). 2021 Aug 28;12(9):1339. doi: 10.3390/genes12091339 (PMC8467453; doi:10.3390/genes12091339)

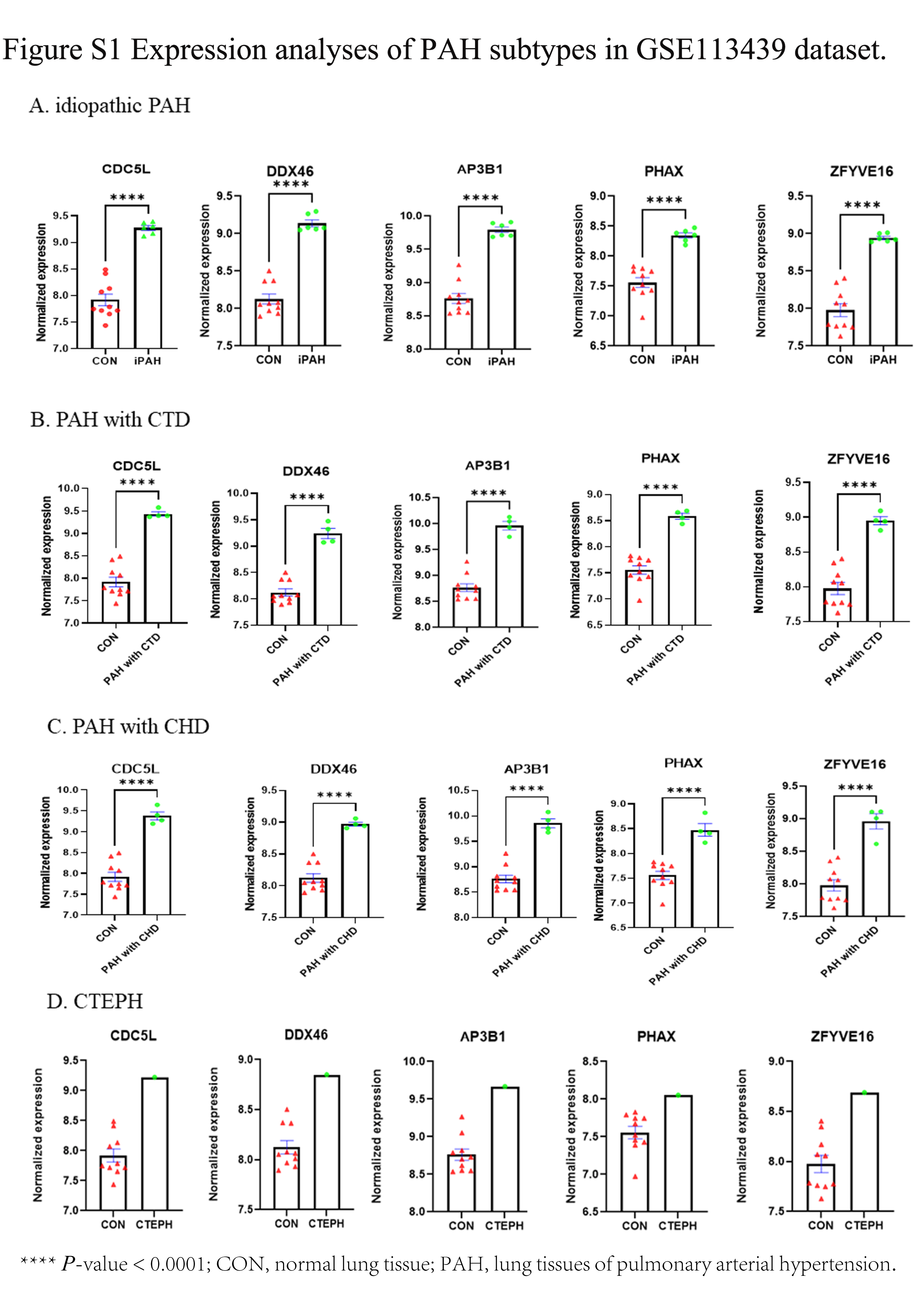

Supplement: Supplementary file 1 [file genes-12-01339-s001.zip › Figure S1.tif]
